# Synchronous orbital and intracranial infantile hemangiomas: a case report and literature review

**DOI:** 10.3389/fped.2026.1799664

**Published:** 2026-04-15

**Authors:** Shuyu Liu, Chi Ma, Cangming Shi, Dawei Chen

**Affiliations:** Department of Neurosurgery, The First Hospital of Jilin University, Changchun, Jilin, China

**Keywords:** cellular infantile hemangioma, centralnervous system, infantile hemangiomas, intracranial infantile hemangiomas, surgery

## Abstract

**Background:**

Solitary orbital and parasellar infantile hemangiomas (IHs) without cutaneous manifestations are exceptionally rare, posing significant diagnostic and therapeutic challenges due to the absence of management consensus.

**Case presentation:**

A 55-day-old female infant presented with progressive left-sided proptosis over the course of one month. Preoperative magnetic resonance imaging (MRI) revealed homogeneously enhancing masses in the left orbit (encasing the optic nerve) and the parasellar region, in which imaging characteristics resembled those of a meningioma. No cutaneous lesions or features of PHACE syndrome were identified. Following multidisciplinary team consultation, a single-stage left pterional approach for the simultaneous resection of both the orbital and parasellar lesions, with the aim of establishing a definitive histopathological diagnosis and relieving mass effect. Histopathological examination confirmed the diagnosis of cellular IH, which could be supported by characteristic morphology and positive immunohistochemical staining for GLUT-1 and CD31. Postoperative recovery was uneventful, with complete resolution of proptosis and no neurological deficits. Imaging confirmed total resection of both lesions.

**Conclusions:**

This case emphasizes that isolated intracranial/orbital IH should be included in the differential diagnosis of pediatric masses, even in the absence of skin involvement. When facing diagnostic uncertainty and significant symptomatology, surgical resection can be a safe and effective primary strategy, providing definitive diagnosis and immediate decompression.

## 1 Introduction

Infantile hemangioma (IH) is the most common non-cancerous vascular tumor found in infants, particularly in women than in men, with a ratio of 3:1 (1). Approximately 60% of cases arise in the head and neck region (2). The classic natural history is characterized by a rapid proliferative phase during the initial postnatal months, followed by a prolonged involution phase typically commencing at the age of one year old (3). Established perinatal risk factors include prematurity, low birth weight, multiple gestation, placental abnormalities, preeclampsia, advanced maternal age, *in vitro* fertilization, and a positive family history (4–6). While the pathogenesis of IH has not been fully elucidated, management strategies involve pharmacological options, such as *β*-blockers, corticosteroids, interferon, and thalidomide, alongside surgical resection and active observation (7–10).

Intracranial extension of IH is exceedingly rare, occurring in less than 0.1% of cases (11). These neuroaxial lesions are mainly associated with PHACE syndrome (Posterior fossa malformations, Hemangiomas, Arterial anomalies, Cardiac defects, and Eye abnormalities) (12). In contrast, solitary intracranial or orbital IH, occurring in isolation without cutaneous manifestations or other syndromic features, is exceptionally rare and remains poorly understood. Consequently, there is a notable absence of consensus regarding the optimal management strategy for these isolated deep lesions, balancing the potential for spontaneous involution against the risks of mass effect and procedural intervention.

An exceptionally rare case of a cellular IH with simultaneous orbital and parasellar involvement in a 55-day-old infant, in the absence of any cutaneous manifestations or features of PHACE syndrome, is presented. The primary objectives of this report are twofold: firstly, to describe the clinical, radiological, and histopathological findings of this unique case, highlighting the challenges associated with preoperative diagnosis and the favorable outcome following surgical resection; and secondly, to provide a comprehensive review of the relevant literature, summarizing the clinical characteristics, diagnostic challenges, and current therapeutic strategies for solitary intracranial and orbital IHs.

## 2 Case presentation

**Figure 1:**
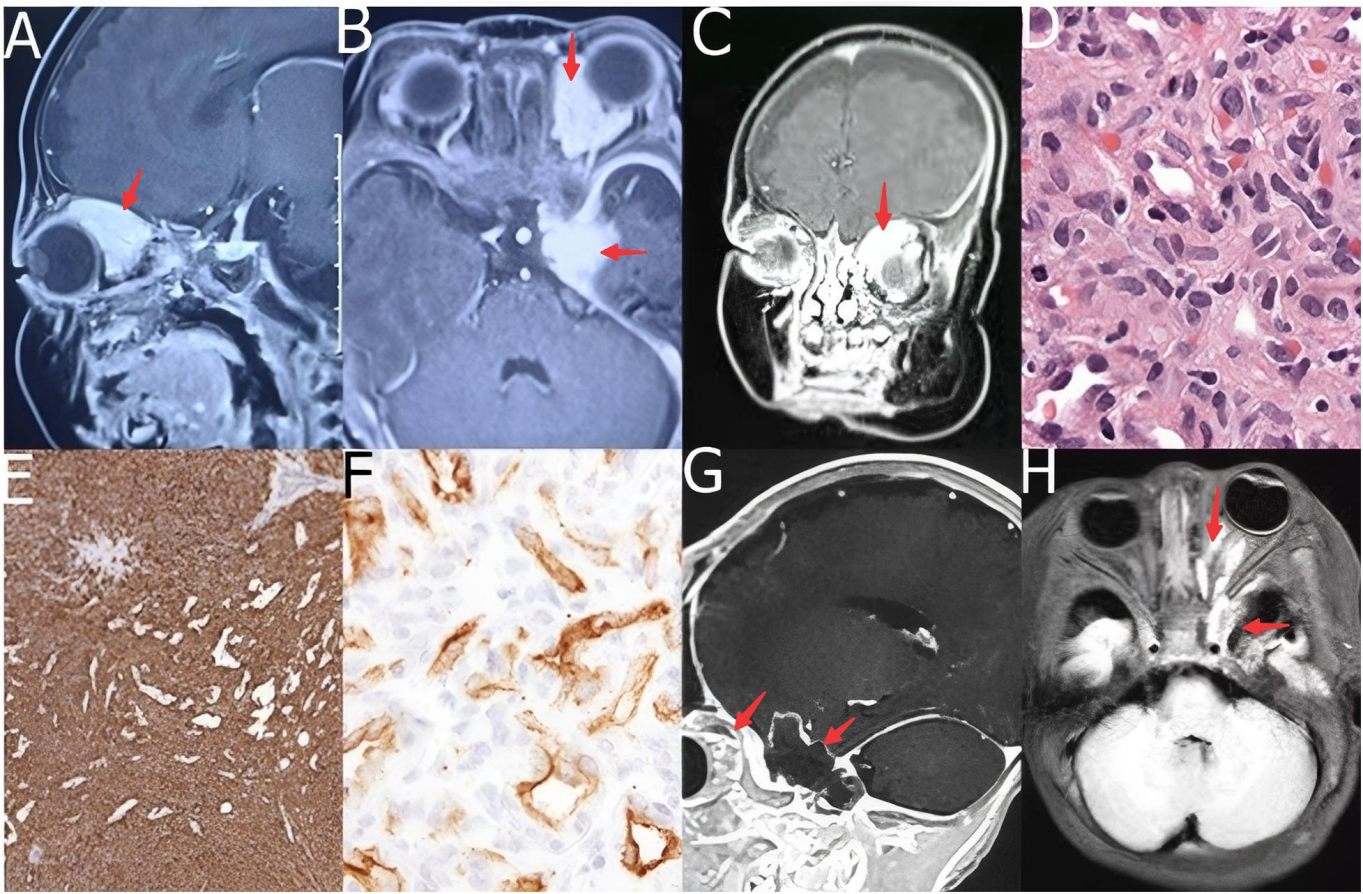
Preoperative imaging, histopathological confirmation, and postoperative outcome of the orbital and parasellar infantile hemangioma. **(A)** Post-contrast orbital MRI showing a homogeneously enhancing lesion (1.6 × 2.0 cm) in the left orbit, encasing the optic nerve and medial rectus muscle. **(B,C)** Post-contrast cranial MRI illustrating a homogeneously enhancing mass (1.5 × 1.8 cm) in the left middle cranial fossa (parasellar region) with dural thickening and enhancement. **(D)** Histopathology (H&E, 20×) depicting a retiform pattern of capillaries surrounded by sheets of immature mesenchymal cells, with occasional mitotic figures (arrow). **(E)** Immunohistochemistry reveals positive CD31 staining (EnVision, 20×). **(F)** Immunohistochemistry showing positive staining for GLUT-1 (EnVision, 20×). **(G)** Postoperative post-contrast MRI indicating complete resection of the orbital lesion. **(H)** Postoperative post-contrast MRI showing complete resection of the parasellar lesion, with only minimal residual dural enhancement.

A 55-day-old female infant was transferred to our hospital due to progressive left-sided proptosis that had been present for one month. She was born at full term via spontaneous vaginal delivery with a birth weight of 3,500 g and had no significant past medical history. Her parents first noted left eye prominence at 25 days of age, which gradually increased and was not worsened with crying. Initial imaging at a local hospital revealed space-occupying lesions in the left orbit and intracranial region, prompting referral to our institution for further evaluation and management. On admission, physical examination showed normal skin without cutaneous vascular lesions. The head circumference was 38 cm (50th percentile), and both the anterior and posterior fontanelles were normotensive and non-bulging. Preoperative ophthalmologic examination demonstrated remarkable left axial proptosis measuring 4 mm. The conjunctiva was clear, without injection or edema. There was no ptosis or evidence of an eyelid hemangioma. Ocular motility was full in all directions of gaze. The pupils were round and equally reactive to light, although a relative afferent pupillary defect (RAPD) was noted in the left eye. Although formal visual acuity testing was limited by the patient's age, the infant exhibited fix-and-follow behavior in both eyes. Fundoscopic examination revealed normal optic discs bilaterally, without evidence of papilledema or optic atrophy. Intraocular pressure was within the normal range. The remainder of the systemic and neurological examination was unremarkable. Preoperative magnetic resonance imaging (MRI) was crucial for evaluation. On T1-weighted sequences, both the orbital and intracranial lesions appeared isointense to the brain parenchyma. On T2-weighted images, both lesions demonstrated uniformly high signal intensity. Following gadolinium administration, the two distinct masses exhibited intense, homogeneous enhancement. The orbital lesion, measuring 1.6 × 2.0 cm^2^, was noted to encase the optic nerve and medial rectus muscle (Figure 1A). The parasellar lesion, measuring 1.5 × 1.8 cm and located in the left middle cranial fossa, was extra-axial with a broad dural base. It demonstrated associated dural thickening and enhancement, aligning with the “dural tail” sign (Figures 1B,C). The initial diagnosis indicated a mass in the left orbital and parasellar regions, with differential diagnoses, including meningioma, and the need to rule out neurofibromatosis type 2 (NF2). Magnetic resonance angiography (MRA) revealed no arterial anomalies, venous malformations, or evidence of vessel encasement. Standard laboratory tests, including complete blood count, urinalysis, serum biochemistry, and cardiac and abdominal ultrasounds, were within normal limits, effectively excluding systemic signs of PHACE syndrome.

A multidisciplinary team (MDT) consultation, involving pediatric neurosurgery, neuroradiology, ophthalmology, pediatric oncology, and anesthesiology was performed. This structured discussion integrated distinct expertise: the neuroradiologist emphasized the meningioma-mimicking imaging features; the ophthalmologist quantified the progressive proptosis and its threat to visual development; the pediatric neurosurgeon assessed the feasibility of gross-total resection based on the lesions' well-circumscribed, extradural anatomy; the pediatric oncologist evaluated the uncertain efficacy and delayed action of first-line propranolol therapy in this setting; and the anesthesiologist addressed perioperative risks specific to infancy. The differential diagnosis included meningioma (potentially NF2-associated), solitary fibrous tumor, or other rare vascular lesions. After synthesizing these perspectives, the consensus favored surgical resection for three key reasons: 1) Diagnostic Uncertainty: The radiographic mimicry of meningioma necessitated histopathological confirmation for definitive diagnosis. 2) Symptomatic Mass Effect: Progressive proptosis indicated active growth posing a direct risk to the optic nerve. 3) Favorable Surgical Anatomy: Both lesions were well-circumscribed and accessible, with the parasellar lesion being extradural, making gross-total resection a feasible and lower-risk objective compared to the uncertain efficacy of medical therapy.

The patient underwent a single-stage surgical procedure via a left pterional approach. The orbital roof was carefully opened, with mobilization of the supraorbital nerve. The periorbita was incised, and orbital fat was gently retracted to access the tumor through the space between the superior and medial rectus muscles. Intraoperatively, the orbital lesion appeared grayish-brown, soft, and highly vascular. After identifying and preserving the optic nerve and ophthalmic vasculature, the tumor was internally debulked to reduce its volume, followed by circumferential dissection and systematic devascularization to achieve complete excision. The parasellar lesion was located in the extradural space, presenting as a grayish-white, well-demarcated, firm, and also highly vascular mass. The intracavernous segment of the internal carotid artery was gently mobilized medially, and the tumor was meticulously dissected along its margins with judicious bipolar coagulation, leading to its *en bloc* removal. Gross-total resection of the two distinct lesions, including one orbital and one parasellar, was successfully achieved, with preservation of the left optic nerve and internal carotid artery. Estimated blood loss was 55 mL, and no transfusion was required.

Histopathological examination of both specimens revealed a similar morphology: a cellular proliferation of immature mesenchymal cells surrounding and interspersed with small, thin-walled capillaries, forming a lobular architecture. The vessels were arranged in a regular retiform pattern. The stroma exhibited moderate cellular density with occasional mitotic figures, while only mild nuclear atypia, being consistent with a proliferative-phase hemangioma (Figure 1D). Immunohistochemistry provided decisive confirmation. Both tumors exhibited strong, diffuse positivity for GLUT-1 and CD31 (Figures 1E,F). The orbital tumor was also positive for Ki-67 (∼20%), SSTR2A (partial), H3K27me3, and S-100. The parasellar tumor exhibited a similar profile with Ki-67 (∼15%) and additional positivity for CD34 and ERG. Staining for markers typically associated with meningioma (EMA, PR), schwannoma (S-100 pattern, SOX-10), or other neural and mesenchymal tumors (STAT6, GFAP, INSM1) was either negative or non-specific. The combined histomorphology and immunophenotype confirmed the final diagnosis of cellular IH.

The patient's recovery following surgery was uneventful. She was monitored in the pediatric intensive care unit for 48 h and received a brief course of dexamethasone to reduce orbital swelling. Significant improvement in the left proptosis was found within 48 h. A postoperative MRI on day 5 confirmed complete resection of both tumors, with no evidence of residual disease (Figures 1G,H). The patient was discharged on postoperative day 10. At the time of discharge, the proptosis had completely resolved. Ophthalmologic and neurological examinations remained unchanged from the preoperative baseline, without new neurological deficits. She remains under long-term outpatient surveillance, with serial clinical and imaging assessments, and has shown no signs of recurrence or neurological deficits to date.

## 3 Discussion and conclusions

The occurrence of IH in the cranial vault represents an extraordinary rarity, posing significant challenges in terms of diagnosis and management due to its obscure etiology, unpredictable pathogenesis, and poorly defined natural history (13). In contrast to their common cutaneous counterparts, intracranial IHs are frequently diagnostically elusive. They are mainly identified incidentally in infants with concomitant surface hemangiomas. While being asymptomatic in numerous instances, their proliferative potential can lead to serious neurological sequelae, including seizures, hydrocephalus, cranial neuropathies, proptosis, and even ischemic or hemorrhagic stroke (7, 14). Orbital IHs frequently lead to eyelid malposition, proptosis, or globe displacement, which can significantly impact visual development by inducing amblyogenic factors, such as strabismus, anisometropia, and deprivation amblyopia. Proptosis, in particular, represents a serious complication, as it may result in exposure keratitis, progressive and irreversible globe protrusion, and optic nerve compression. According to Tambe et al.'s research, 17% of hemangiomas are located in the orbital region and may extend into the orbit, leading to proptosis or displacement of the globe (15). Haik et al. reported that 7% of periocular hemangiomas involve the deep orbit, resulting in proptosis, exposure keratitis, and optic nerve compression (16). PHACE syndrome is frequently associated with retro-orbital IH and proptosis. In a prospective study of 1,096 IH patients conducted by Metry et al., 25 were diagnosed with PHACE syndrome, 20% of whom exhibited retro-orbital involvement, and all affected infants had cutaneous IH (14). highlighting the importance of excluding PHACE syndrome or intracranial hemangioma in cases of infantile proptosis (17, 18).

A systematic review of the literature, as summarized in Table 1, revealed instructive patterns among the scant reports of surgically managed intracranial IH. Analysis of the 12 documented cases (including ours) indicated a strong predilection for the very young infant, with the majority presenting within the first 4 months of life (19–28). Crucially, none of these surgically treated cases were associated with PHACE syndrome, demonstrating that isolated, symptomatic intracranial IH may represent a distinct clinical entity necessitating intervention. The present case aligns with these patterns in terms of its early symptomatic presentation, while it is distinguished by its unique dual-compartment involvement (orbit and parasellar region) in a patient as young as 55 days.

**Table 1:** Recorded cases of intracranial infantile hemangiomas who were treated surgically (1993–2025).

Author	Age/gender	Neurological or Ophthalmic Complaint	Intracranial Location of Hemangioma	Outcome
Willing et al. (19)	17 months/male	Seizures, developmental delay	Right temporal dura	Resolution
Karikari et al. (20)	3 months/ male	Central hypotonia	Fourth ventricle, left CPA	Resolution
Daenekindt et al. (21)	7 weeks/male	Enlarged head circumference	Right temporal fossa	Resolution
Uyama et al. (22)	4 months/female	Hydrocephalus	Left cerebellar hemisphere	Resolution
Philpott et al. (23)	12 months/female	head circumference Enlarged	Dura of right parietal lobe	Resolution
Zheng et al. (24)	3 years/male	Somnolence, right CNIII palsy	Middle cranial fossa	Resolution
Jalloh et al. (25)	2 weeks, Male	enlarging head circumference seizures	Left middle cranial fossa	Resolution
Shakir et al. (26)	2 weeks, female	Hydrocephalus	Posterior fossa	Resolution
Dalsin et al. (23)	37 weeks gestation	female Diagnosed on antenatal ultrasound	Left middle cranial fossa	Resolution
Haine et al. (27)	3 weeks, male	Symptoms of raised ICP	Posterior fossa	Resolution
Albalawi et al. (28)	4 weeks, male	vomiting and head circumference increase	right frontoparietal region	Resolution
Present Case, 2025	55 days/Female	Progressive proptosis	Left orbit &parasellar (middle fossa)	Resolution

GA, gestational age; US, ultrasound; ICP, intracranial pressure; CP, Cerebellopontine; CN, cranial nerve.

MRI scans of infantile intracranial hemangiomas are non-specific, typically presenting as well-defined lesions with isointense signals on T1-weighted images, hyperintense signals on T2-weighted images, and significant contrast enhancement. In cases without associated cutaneous IHs, differential diagnosis must include other intracranial tumors, such as meningiomas, cavernous hemangiomas, and hemangioblastomas (11, 29).

However, histopathology remains the gold standard for diagnosis. The observed morphology, lobular, GLUT-1-positive capillary proliferations interspersed in immature mesenchyme, was pathognomonic for cellular IH (24, 25). The upregulation of GLUT-1, a key glucose transporter, is mechanistically linked to the high metabolic demand and proliferative drive of IH endothelial cells during the rapid growth phase, serving as a reliable molecular hallmark of active angiogenesis. The immunohistochemical profile was conclusive: strong, diffuse positivity for GLUT-1 and CD31, alongside the absence of markers typically associated with meningothelial (EMA, PR), schwannian (diffuse S-100/SOX-10), and STAT6 markers, effectively excluded meningioma, schwannoma, and solitary fibrous tumor, respectively. Importantly, this GLUT-1 positivity also helps distinguish IH from other vascular lesions such as vascular malformations and cavernous hemangiomas, which are typically GLUT-1 negative, thereby reinforcing its diagnostic specificity. This case highlights the critical role of GLUT-1 immunostaining in confirming IH in the central nervous system, converting a radiological mimic into a definitive diagnosis.

The optimal management strategy for orbital and intracranial IH lacks consensus, necessitating a highly individualized approach based on symptomatology, anatomical location, and diagnostic certainty. Surgical resection may be considered as the primary management strategy in the following scenarios: (1) when a definitive histopathological diagnosis is required due to radiologic ambiguity, as in the present case, in which meningioma was the leading differential diagnosis; (2) in the presence of significant or progressive mass effect, including elevated intracranial pressure with focal neurological deficits, or rapidly enlarging intraconal lesions causing proptosis, eyelid malposition, or optic nerve compromise; (3) when the lesions are surgically accessible with an acceptable risk profile; and (4) when gross-total resection is feasible and represents a lower-risk option compared with the uncertain efficacy and delayed therapeutic response of medical therapy. In carefully selected complex cases, a transcranial approach possesses several advantages: it provides a definitive pathological diagnosis, achieves immediate decompression of mass effect, relieves focal neurological symptoms, and prevents further neurological deterioration. Moreover, early surgical intervention increases the likelihood of achieving gross-total resection while avoiding the potential delays and treatment failures associated with observation or pharmacologic therapy.

The therapeutic landscape of IH was transformed in 2008 following the seminal report by Léauté-Labrèze et al., demonstrating the efficacy of propranolol for severe cutaneous IH. This discovery regarded propranolol, a non-selective β-ocker, as the first-line therapy for high-risk IH, a recommendation later formalized by the American Academy of Pediatrics (AAP) (30, 31). However, management of the exceedingly rare orbital and intracranial IH remains poorly defined, lacking evidence-based guidelines. Emerging clinical experience demonstrates that propranolol may be effective in this setting, although evidence is limited. Its efficacy in deep intracranial lesions may differ from that in cutaneous IH due to factors, such as blood-brain barrier penetration, distinct hemodynamics, and potentially different proliferative kinetics of neuraxial endothelial cells. Elise et al. documented complete regression in four of five cases of intracranial IH treated with oral propranolol (13), supporting the consideration of a therapeutic trial of propranolol as a strategy to avoid high-risk biopsy or surgical intervention, particularly in asymptomatic lesions where spontaneous involution may still occur (32). This approach contrasts with surgical resection prioritized for definitive diagnosis or to address significant mass effect, as illustrated in the present case. Other pharmacological agents (e.g., corticosteroids, interferon, and thalidomide) play a limited role and are typically reserved for complex cases that are refractory to first-line therapy.

Regarding long-term outcomes, resection of orbital and parasellar lesions in infants warrants careful monitoring for potential sequelae. These include, but are not limited to, visual pathway dysfunction, neuroendocrine disturbance due to parasellar/sellar manipulation, and impacts on neurocognitive development. The favorable anatomical preservation achieved in this case, along with the benign nature of IH, suggests a low risk of such complications, underscoring the importance of meticulous surgical technique and structured multidisciplinary follow-up in optimizing long-term neurological and developmental outcomes.

This study has inherent limitations, primarily arising from its nature as a single-case report. Although follow-up is ongoing, the duration remains intermediate, and the long-term prognosis has yet to be fully established. Additionally, the technical success achieved in this case may not be generalizable to all centers, particularly those lacking dedicated pediatric skull base expertise.

## Data Availability

The raw data supporting the conclusions of this article will be made available by the authors, without undue reservation.
